# Posthospital Fall Injuries and 30-Day Readmissions in Adults 65 Years and Older

**DOI:** 10.1001/jamanetworkopen.2019.4276

**Published:** 2019-05-24

**Authors:** Geoffrey J. Hoffman, Haiyin Liu, Neil B. Alexander, Mary Tinetti, Thomas M. Braun, Lillian C. Min

**Affiliations:** 1 Department of Systems, Populations and Leadership, University of Michigan School of Nursing, Ann Arbor; 2 Institute for Healthcare Policy and Innovation, University of Michigan, Ann Arbor; 3 Geriatric Research Education and Clinical Care Center (GRECC), VA Medical Center, Ann Arbor, Michigan; 4 Division of Geriatric and Palliative Medicine, Department of Medicine, University of Michigan, Ann Arbor; 5 Division of Geriatrics, Department of Internal Medicine, Yale School of Medicine, New Haven, Connecticut; 6 School of Public Health, Yale University, New Haven, Connecticut; 7 Department of Biostatistics, University of Michigan School of Public Health, Ann Arbor; 8 Veterans Affairs Center for Clinical Management and Research (CCMR), VA Medical Center, Ann Arbor, Michigan; 9 Institute for Social Research, University of Michigan, Ann Arbor

## Abstract

**Importance:**

Falls are common among older adults, particularly those with previous falls and cognitive impairment and in the postdischarge period. Hospitals have financial incentives to reduce both inpatient falls and hospital readmissions, yet little is known about whether fall-related injuries (FRIs) are common diagnoses for 30-day hospital readmissions.

**Objective:**

To compare fall-related readmissions with other leading rehospitalization diagnoses, including for patients at greatest risk of readmission.

**Design, Setting, and Participants:**

Retrospective cohort study of the Hospital Cost and Utilization Project’s Nationwide Readmissions Database of nationally representative US hospital discharges among Medicare beneficiaries aged 65 years and older from January 1, 2013, to November 30, 2014. The prevalence and ranking of FRIs compared with other diagnostic factors for 30-day unplanned hospital-wide readmissions were determined, overall and for 2 acute geriatric cohorts, classified by fall injury or cognitive impairment diagnoses observed at the index admission. Analyses were also stratified by patient discharge disposition (home, home health care, skilled nursing facility). Analyses were conducted from February 1, 2018, to February 26, 2018.

**Main Outcomes and Measures:**

Unplanned hospital-wide readmission within 30 days of discharge.

**Results:**

From the database, 8 382 074 eligible index admissions were identified, including 746 397 (8.9%) in the FRI cohort and 1 367 759 (16.3%) in the cognitive impairment cohort. Among the entire 8 382 074-discharge cohort, mean (SD) age was 77.7 (7.8) years and 4 736 281 (56.5%) were female. Overall, 1 205 962 (14.4%) of index admissions resulted in readmission, with readmission rates of 12.9% for those with a previous fall and 16.0% for patients with cognitive impairment. Overall, FRIs ranked as the third-leading readmission diagnosis, accounting for 60 954 (5.1%) of all readmission diagnoses. Within the novel acute geriatric cohorts, FRIs were the second-leading diagnosis for readmission both for patients with an FRI at index admission (10.3% of all readmission diagnoses) and those with cognitive impairment (7.0% of all readmission diagnoses). For those with an FRI at index admission and discharged home or to home health care, FRIs were the leading readmission diagnosis.

**Conclusions and Relevance:**

This study found that posthospital FRIs were a leading readmission diagnosis, particularly for patients originally admitted with a FRI or cognitive impairment. Targeting at-risk hospitalized older adults, particularly those discharged to home or home health care, is an underexplored, cost-effective mechanism with potential to reduce readmissions and improve patient care.

## Key Points

**Question:**

To what extent do falls play a role in hospital readmissions for older patients, including those with acute geriatric risk factors?

**Findings:**

This cohort study using Hospital Cost and Utilization Project data from 8.3 million Medicare beneficiaries found that fall-related injuries ranked as high as the third-leading readmission diagnosis, depending on the type of initial hospitalization. Fall injuries ranked still higher for patients with a high preexisting risk of falling and for those discharged home or to home health care rather than to a skilled nursing facility.

**Meaning:**

Fall-related injuries are leading diagnoses for hospital readmissions, particularly for at-risk older adults discharged home, highlighting the need for greater attention to transitional prevention strategies to avoid postdischarge falls.

## Teaser

This cohort study of data from the Hospital Cost and Utilization Project Nationwide Readmissions Database compares 30-day fall-related readmissions with other leading rehospitalization diagnoses in Medicare beneficiaries 65 years and older with a variety of index admissions.

## Introduction

Despite effective tools for their prevention, falls remain common and costly among older adults,^1^ with recently discharged patients^2,3^ and those with cognitive impairment at greatest risk.^4^ Although Medicare incentivizes attention to inpatient safety with both its “never events”^5^ and readmission policies,^6^ the importance of falls for rehospitalization has not been examined. Hospitals have increased their focus on fall prevention after the introduction of the Hospital-Acquired Conditions program and on care transitions, including the management of patient discharge in the context of preventing readmissions,^7,8,9^ under the strong incentives of the Hospital Readmissions Reduction Program (HRRP).^12,13,14^

Whether falls contribute to readmission prevalence, which can increase hospital penalties under HRRP, and whether they are more common for older patients presenting with a fall-related injury (FRI) or with cognitive impairment is not known. Studies from 1994^2^ and 2000^3^ observed increased patient fall rates after hospital discharge, suggesting a heightened risk for fall-related readmission, but these studies relied on data from small, non–nationally representative patient samples. A number of studies have identified disability, chronic illness, functional limitation, and cognitive impairment as factors for readmission,^7,8,9,10^ and all of which are separately associated with risk of falling.^4^ Falls are the leading cause of injury-related hospitalization among older adults^11^ and, as evidenced in the qualitative literature, there are worrisome gaps in evidence-based fall prevention for patients recently discharged from the hospital.^12,13,14^ It may be that poor cognitive and functional recovery after a hospitalization increases readmission risk, particularly for patients with preexisting functional and cognitive impairment. For patients with multiple impairments, such as previous FRIs in addition to cognitive issues, these risks may be compounded, particularly when postdischarge guidance is limited. However, although a seminal study by Jencks et al^15^ identified leading causes for readmission, including heart failure and septicemia, that study and others have not thoroughly assessed the association between readmission and FRIs among older adults, including those with functional and cognitive risk factors at the index admission.

In this study, we aimed to evaluate whether falls are a prevalent diagnosis for hospital readmissions for older Medicare beneficiaries, including those presenting at the index admission with an FRI and those with cognitive impairment. Our objectives were to understand how FRIs figured among the leading diagnoses at readmission for patients with varying diagnoses at index admission and whether we could use index admission and discharge data to identify patients at high risk for postdischarge falls. With this approach, our findings will provide policy makers and hospital discharge staff with a better understanding of the prevalence of a common and preventable factor for readmission among at-risk older adults.

## Methods

### Data Sources and Study Population

This study followed the Strengthening the Reporting of Observational Studies in Epidemiology (STROBE) reporting guideline. We used hospital discharge data from the Nationwide Readmissions Database (NRD), a data set that is part of the Agency for Healthcare Research and Quality’s Hospital Cost and Utilization Project. The NRD contains nationally representative data obtained from the Hospital Cost and Utilization Project’s state inpatient databases and allows for patient records linkages to examine readmissions. We used discharge data from the 2013 and 2014 NRDs. This study was approved by the Institutional Review Board at the University of Michigan. The nature of this study did not require informed consent from study participants. The Hospital Cost and Utilization Project NRD is a limited data set that contains no direct patient identifiers. Analyses were conducted from February 1, 2018, to February 26, 2019.

### Study Population

Our initial study population was an eligible full index admission cohort that included discharges of Medicare fee-for-service and Medicare Advantage beneficiaries aged 65 years and older. Using Centers for Medicare & Medicaid Services criteria for all-cause hospital-wide readmission (HWR), we excluded index admissions involving an in-hospital death; patient transfer to another acute care facility; patients with fewer than 30 days’ follow-up discharge data for the index discharge (ie, with index admissions occurring after November 30 of each of the years 2013 and 2014, given that the NRD does not allow for cross-year patient linkages); and patients discharged against medical advice, for primary psychiatric diagnoses, for rehabilitation, and for cancer.^16^

Beginning with the full index admission cohort (eTable 1 in the Supplement), we developed 2 additional index cohorts using primary or secondary diagnoses and procedure codes (*International Classification of Diseases, Ninth Edition *codes) from the index admission.^16^ These were novel index admission inpatient cohorts with geriatric conditions, patients treated for an FRI and those with evidence of cognitive impairment or delirium at the index admission. These cohorts were not mutually exclusive. We also stratified by patient discharge disposition (routine discharge to home, home health care, and other transfer—including transfer to a skilled nursing or intermediate care facility) to examine whether readmission diagnoses varied by type of discharge.

As a sensitivity analysis, we followed the original method developed by Centers for Medicare & Medicaid Services to study HWR mechanisms, an approach that stratifies patients into 5 mutually exclusive index admission clinical cohorts: medicine, cardiorespiratory, cardiovascular, neurology, and surgery.^16^

Falls were defined using an adapted accurate algorithm^1,17,18,19^ that uses any primary and secondary diagnostic *ICD-9* codes and external cause-of-injury codes (e-codes E880, E881, E882, E883, E884, E885, E886, E888) to identify injuries by body type (upper extremity, lower extremity, head and face, neck and trunk). E-codes recorded the mechanism of the injury.^20^ We excluded e-coded injuries that were not plausibly fall related (eg, E812, E813, E814, which are motor vehicle traffic crashes).

Cognitive impairment included both acute changes to cognition (delirium, amnesia) and chronic conditions (dementia, cognitive disorders) using the Agency for Healthcare Research and Quality’s Clinical Classification Software code 653 from the index admission.^21^ Comorbidities at the index admission were identified using a count of Clinical Classification Software codes.

After identifying these index admission cohorts, we developed study subsamples that included only readmissions, for the purposes of exploring the types of diagnoses, including FRIs, that were coded at the readmission visit. These subsamples involved only observations that were readmissions for patients in the full index admission cohort and in each of the 2 index admission acute geriatric cohorts. In the sensitivity analysis, we explored readmission diagnoses for patients in each of the 5 clinical cohorts (eTables 1-5 in the Supplement).

### Primary Outcome: Prevalence and Ranking of FRIs as Readmission Diagnoses

We explored the prevalence and ranking of FRIs compared with other diagnoses for 30-day unplanned HWRs.^16^ To do so, we categorized the diagnosis at the rehospitalization following the methods of Jencks et al,^15^ which used mutually exclusive groupings of Medicare diagnosis related groups (DRGs, version 31) associated with a readmissions claim. Diagnosis related groups are categories of patient care involving clinically similar diagnoses that require similar amounts of resources. They are relevant for readmissions because HRRP penalties are taken from each of a hospital’s DRG payments for hospitals with excess readmissions.^22^ Per the Centers for Medicare & Medicaid Services’ HWR methods, planned readmissions, including maintenance chemotherapy, rehabilitation, hip and knee replacements, and skin grafts, were excluded.^16,21^ In addition, a readmission that met the eligibility criteria could also be included itself as part of the index admission cohort.^16^

Because most FRIs are not identifiable using DRGs, we defined an FRI readmission as any FRI diagnosis (primary or secondary) on the readmission visit using the same diagnostic codes as the above index admission. The diagnostic injury codes were divided into 4 anatomic regions (head/face, neck/trunk, upper extremity, lower extremity), and generalized fall (e-coded fall without a diagnosis mappable to an anatomic region). If the index admission was an FRI admission, any injury within the same anatomic region as the index admission was ineligible as an FRI readmission. Similarly, any generalized FRI at readmission preceded by a generalized FRI that involved the same e-code was excluded as an FRI readmission. These last 2 exclusions were designed to avoid potentially double-counting treatment for the same injury, which has been noted as a concern in the literature.^18,19^ In total, these 2 exclusions represented approximately 2% of FRI readmissions, resulting in a more conservative count of FRI readmission diagnoses.

For clarity, we distinguish FRIs identified at the index admission from subsequent readmission using the terms *index FRI admission* and *FRI readmission*; similarly, we reference a patient with an FRI occurring during these visits as an *index faller*, *readmission faller*, or both.

### Statistical Analysis

Our first step was to examine the sociodemographic and clinical characteristics of patients in the full index admission cohort and then among index fallers, using χ^2^ tests to compare proportions and *t* tests to compare means. Two-tailed *P* < .05 was considered statistically significant. We also examined the top 10 Clinical Classification Software diagnoses for fallers compared with nonfallers. Our next step was to describe readmission rates overall and for the 2 index admission acute geriatric cohorts. We also report readmission rates stratified across 3 patient discharge dispositions: routine discharge to home, home health care, and nonhome transfers. The last category consists of transfers to postacute skilled nursing facilities and intermediate care facilities, both of which involve skilled nursing. Transfers to hospitals for short-term stays were included in the nonhome transfers category, which were discharge dispositions for approximately 1% of all discharges. Approximately 0.1% of discharges had missing or invalid values for discharge disposition. Analyses were performed using SAS software version 9.4 (SAS Institute, Inc),

We then narrowed our study sample to only our 3 subsamples of patients with an unplanned HWR. Beginning with the subsample of patients from the full index admission cohort who were readmitted, we computed the percentage of readmissions with an FRI diagnosis and where FRIs, compared with all other DRGs, ranked among all diagnoses at the readmission visit (ie, the relative proportion of all readmissions with a specific diagnosis). We then separately repeated these 2 steps for readmitted patients from each of the 2 index admission acute geriatric cohorts and stratified by patient discharge disposition. In sensitivity analyses, we report these rankings for the 2 acute geriatric cohorts in combination with the original 5 clinical cohorts.

## Results

We identified 8 382 074 eligible index admissions for older Medicare beneficiaries from January 1 to November 30, 2013, and January 1 to November 30, 2014. Among the entire discharge cohort of 8 382 074 individuals, the mean (SD) age was 77.7 (7.8) years and 4 736 281 (56.5%) were female. Table 1 presents characteristics of patients overall and for index fallers, who composed 746 397 (8.9%) of all index admissions. Of the 8 382 074 index admissions, 1 367 759 (16.3%) involved the cognitively impaired index admission acute geriatric cohort. As expected, the index fallers were older than nonfallers, and 201 665 (27.0%) also had cognitive impairment, compared with 1 166 094 of the 7 635 677 nonfallers (15.3%). However, the mean (SD) comorbidity score was lower for index fallers than nonfallers (16.9 [14.6] vs 17.9 [15.5]); leading Clinical Classification Software diagnoses for index fallers often involved fractures and superficial injuries, whereas nonfallers were commonly diagnosed with chronic conditions (eTable 2 in the Supplement). Substantially more fallers than nonfallers were transferred to skilled nursing facilities (Table 1).

**Table 1.** Sociodemographic and Clinical Characteristics of Medicare Beneficiaries 65 Years and Older Overall and by Fall-Related Injury Status for US Hospital Discharges, 2013-2014

Characteristic	Overall (N = 8 382 074)	Fallers (n = 746 397)	Nonfallers (n = 7 635 677)	*P* Value^a^
Age, y, No. (%)				
65-74	3 269 688 (39.0)	182 289 (24.4)	3 087 399 (40.4)	<.001
75-84	3 056 786 (36.5)	268 712 (36.0)	2 788 074 (36.5)	
≥85	2 055 600 (24.5)	295 396 (39.6)	1 760 204 (23.1)	
Female, No. (%)	4 736 281 (56.5)	490 639 (65.7)	4 245 642 (55.6)	<.001
Cognitive impairment, No. (%)	1 367 759 (16.3)	201 665 (27.0)	1 166 094 (15.3)	<.001
Comorbidity score, mean (SD)	17.8 (15.4)	16.9 (14.6)	17.9 (15.5)	<.001
Discharge disposition, No. (%)				
Routine	3 957 102 (47.2)	160 254 (21.5)	3 796 848 (49.7)	<.001
Skilled nursing facility	2 468 912 (29.5)	442 194 (59.2)	2 026 718 (26.5)	
Home health care	1 862 288 (22.2)	133 737 (17.9)	1 728 551 (22.6)	
Other	93 772 (1.1)	10 212 (1.4)	83 560 (1.1)	

^a^ Compares fallers with nonfallers. χ^2^ tests were used to compare proportions. Two-sample *t* tests were used to compare means. Falls were assessed at the index discharge.

For those in the full index admission cohort, the rate of unplanned HWRs was 16.3% (Table 2). Compared with nonfallers, index fallers paradoxically had a 12% lower readmission rate (14.5% vs 12.9%, respectively; *P* < .001) (Table 2). This difference was driven by the largest index admission clinical cohort, medicine, in which nonfallers had an approximately 20% greater readmission rate than index fallers (16.3% vs 13.4%; *P* < .001) (eTable 3 and eTable 4 in the Supplement). As expected, patients with cognitive impairment had 13% higher readmission rates relative to those without impairment (16.0% vs 14.0%; *P* < .001) (Table 2). Readmissions were more common for patients discharged to skilled nursing facilities (17.1%) or who received home health care (16.4%) than those with routine discharges to home (11.7%).

**Table 2.** Unplanned 30-Day All-Cause Readmission Prevalence Among Medicare Beneficiaries 65 Years and Older Overall and by Acute Geriatric Cohort, Overall and by FRI Status, 2013-2014

Discharge Characteristic	Overall Readmission (N = 8 382 074)	Index FRI Acute Geriatric Cohort Readmission		*P* Value^a^	Index Cognitive Impairment Acute Geriatric Cohort Readmission		*P* Value^a^
		Yes (n = 746 397)	No (n = 7 635 677)		Yes (n = 1 367 759)	No (n = 7 014 315)	
All discharges, No. (%)	1 205 962 (14.4)	96 301 (12.9)	1 109 661 (14.5)	<.001	218 351 (16.0)	987 611 (14.0)	<.001
Discharge Disposition, No. (%)							
Routine (n = 3 957 102)	464 527 (11.7)	17 098 (10.7)	447 429 (11.8)	<.001	43 107 (14.5)	421 420 (11.5)	<.001
Skilled nursing facility (n = 2 468 912)	421 033 (17.1)	59 763 (13.5)	361 270 (17.8)	<.001	124 858 (16.2)	296 175 (17.4)	<.001
Home health care (n = 1 862 288)	304 544 (16.4)	17 772 (13.3)	286 772 (16.6)	<.001	47 629 (16.8)	256 915 (16.3)	<.001
Other (n = 93 772)	15 858 (16.9)	1668 (16.3)	14 190 (17.0)	.10	2757 (15.1)	13 101 (17.3)	<.001

Abbreviation: FRI, fall-related injury.

^a^ χ^2^ tests were used to compare proportions.

Examining only 1 205 962 readmissions among the full index admission cohort, FRIs were ranked third (ie, FRIs as a proportion of readmission diagnoses was third-highest among all diagnoses) at 5.1% (60 954), after only septicemia (115 026 [9.5%]) and heart failure (105 771 [8.8%]) (Table 3). Among the 96 301 readmissions for only patients in the acute geriatric fall cohort (index fallers), FRI diagnoses were twice as common (9915 [10.3%]). They ranked second, trailing only septicemia (10 079 [10.5%]) as a leading diagnosis. Among 218 351 readmissions for only patients in the cohort with cognitive impairment on index admission, FRIs were also the second-leading diagnosis at readmission (15 262 [7.0%]) (Table 3).

**Table 3.** Percentage of 30-Day Unplanned All-Cause Readmissions of Medicare Beneficiaries 65 Years and Older Overall and Among FRI and Cognitive Impairment Index Cohort, 2013-2014^a^

Rank	Diagnosis (No. [%])		
	Overall Readmissions (N = 1 205 962)	FRI Cohort Readmissions (n = 96 301)	Cognitive Impairment Readmissions (n = 218 351)
1	Septicemia (155 026 [9.5])	Septicemia (10 079 [10.5])	Septicemia (30 972 [14.2])
2	Heart failure (105 771 [8.8])	FRI (9915 [10.3])	FRI (15 262 [7.0])
3	FRI (60 954 [5.1])	Heart failure (5397 [5.6])	Heart failure (14 239 [6.5])
4	Respiratory (56 460 [4.7])^b^	Respiratory (4205 [4.4])^b^	Respiratory (13 270 [6.1])^b^

Abbreviation: FRI, fall-related injury.

^a^ This table presents the rankings of the leading diagnoses for readmission and the proportion of all readmissions represented by a given diagnosis (eg, septicemia was the leading overall readmission diagnosis, representing 9.5% of all readmission diagnoses).

^b^ Respiratory or ventilation issues.

The ranking of FRIs as readmission diagnoses varied by patient discharge disposition (Table 4). Although FRIs were the third-leading readmission diagnosis for patients discharged to home health care (16 324 of 304 544 [5.4%]), they were the fourth-leading diagnosis for those transferred to a skilled nursing facility (23 157 of 421 033 [5.5%]) and fifth-leading diagnosis for those routinely discharged home (20 754 of 464 527 [4.5%]). Fall-related injuries were more common readmission diagnoses for patients in the acute geriatric fall cohort who were discharged home (2103 of 17 098 [12.3%]) or to home health care (2091 of 17 772 [11.8%]), compared with those transferred to a skilled nursing facility (5479 of 17 098 [9.2%]). Fall-related injuries were less common readmission diagnoses for patients readmitted from the cognitive impairment cohort who were discharged home (3189 of 43 107 [7.4%]) and to home health care (3328 of 47 629 [7.0%]) compared with a skilled nursing facility transfer (8574 of 124 858 [6.9%]).

**Table 4.** Ranking of FRI Among Top 30-Day Unplanned All-Cause Readmission Diagnoses for Medicare Beneficiaries 65 Years and Older by Patient Discharge Disposition, 2013-2014

Cohort	Discharge Disposition, Rank (FRI Readmissions, No./All Readmissions, No. [%])			
	Routine	Skilled Nursing Facility	Home Health Care	Other^a^
Overall	5 (20 754/464 527 [4.5])	4 (23 157/421 033 [5.5])	3 (16 324/304 544 [5.4])	4 (719/15 858 [4.5])
FRI index cohort	1 (2103/17 098 [12.3])	2 (5479/59 763 [9.2])	1 (2091/17 772 [11.8])	1 (242/1668 [14.5])
Cognitive impairment cohort	3 (3189/43 107 [7.4])	2 (8574/124 858 [6.9])	3 (3328/47 629 [7.0])	3 (171/2757 [6.2])

Abbreviation: FRI, fall-related injury.

^a^ Includes short-term hospital discharge dispositions.

In sensitivity analyses, among all readmissions, FRIs were the third-leading diagnosis for patients in the medicine (31 161 of 570 530 [5.5%]) and neurology (5046 of 63 635 [7.9%]) cohorts (eTable 5 in the Supplement). For index fallers, FRIs ranked as the leading readmission diagnosis for patients in the medicine (4962 of 41 111 [12.1%]) and neurology (1531 of 10 201 [15.0 %]) cohorts and second for those in the cardiovascular (417 of 4368 [9.6%]) and surgery (2429 of 33 913 [7.2%]) cohorts.

## Discussion

Fall-related injuries are leading diagnoses for hospital readmissions. Unsurprisingly, their role in readmissions was greatest for those with an index FRI or cognitive impairment. To avoid penalties and improve care, postdischarge fall prevention protocols should be investigated as a new way to prevent readmissions, especially among the patients in these acute geriatric cohorts. Older patients with a previous fall, cognitive impairment, or both may need more specific, targeted interventions than those they are at present receiving.

To put our findings into perspective, if an intervention could reduce FRI readmissions among all discharged patients by 20% in our sample of 1.2 million US nationwide readmissions across 2 years, an estimated 12 000 of 60 000 FRI readmissions could potentially be avoided. This translates to 1% of all readmissions. Given the observed overall 14.4% all-cause HWR rate (which was consistent with published rates from the same period),^23^ 1% absolute risk reduction in readmissions would represent a small but measurable difference, because even small decreases in readmissions can substantially lower Medicare hospital penalties (1 avoided readmission can result in savings of ≥$10 000, according to recent research).^24^

Previous work has identified heart failure,^15^ hospital-acquired conditions, and other patient safety indicators^22,23,24,25,26^ as leading causes of readmission. The present study suggests falls as a diagnosis of equal or greater importance for readmission,^3^ suggesting a need for greater focus on fall prevention during and after hospitalization,^13,25,26^ particularly among patients treated for a fall at the index discharge and who are then discharged home. Existing research suggests that patients and their caregivers are often unaware of evidence-based best practices when they leave the hospital and that they could benefit from enhanced transitional fall prevention.^14,26^ Such prevention might boost the benefits of existing readmission prevention models.^27,28,29,30^ Moreover, finding posthospital falls a common diagnosis in readmissions suggests the unintended consequences of another Medicare quality program: Medicare’s “never events” program, which penalizes hospitals for the occurrence of inpatient fall injuries. Because high-risk patients are labeled as fallers on admission, they are discouraged from getting up and moving without assistance or subjected to technologies such as alarms and robots to enforce immobility. This immobility is associated with an increased risk for falls.^5,31,32^

Our finding that risks of postdischarge falls were greatest for older adults with prior falls and cognitive impairment is also consistent with findings reported by other investigators.^4,33,34^ The finding that patients discharged home or to home health care, compared with a skilled nursing facility, may be at greater risk for an FRI readmission is a novel one, potentially indicating gaps in care for certain patients. It also suggests, along with more recent evidence on hospital readmission risk and postacute care, a protective benefit from skilled nursing care.^35^ Taken together, the findings suggest a role for targeted fall prevention in readmissions, as these higher-risk patients may be particularly decompensated from immobility or fatigue, or lacking in fall self-awareness or access to prevention interventions^13,25,36^ after a hospital stay. Furthermore, they may not receive adequate posthospital supervision, assistance with self-care tasks, nursing surveillance, or rehabilitation services when discharged home. In response, clinicians can identify at-risk patients using common screenings for fall risk and cognitive status among hospitalized patients^37,38^ and implement multifactorial fall preventions that are associated with risk reductions of approximately 20%.^39^ Such interventions include both hospital-side and posthospital activities such as rehabilitation, medication review, use of assistive devices, home modification, and strength and flexibility training. Enhanced transitional training for patients discharged home and their family caregivers may also be warranted.^14^ These potential lessons for improving care are broadly generalizable to the US older adult population, given that the study analyzed data of both fee-for-service and Medicare Advantage beneficiaries.

### Limitations

Our study has several limitations. First, the analysis comparing rankings of DRG-based and FRI readmissions is not “apples to apples,” because the FRI readmission and the DRG-based categories are not mutually exclusive; however, we would expect that this would misclassify the FRI- vs DRG-based reasons toward similar rankings if a large number of patients with FRIs were admitted to a single DRG-based category. In all, our estimates of fall-related readmissions are likely conservative, given that DRGs are reimbursed but fall e-codes are not; therefore, many falls are likely unreported in the claims data. Our estimates of fall-related readmission diagnoses are also conservative given that we did not count same-injury diagnoses that occurred within 30 days of one another to avoid double-counting multiple treatments for the same injury. Also, although we used the UCLA/RAND algorithm,^18,19^ identifying FRIs with hospital claims data does not perfectly capture all FRIs. To improve its accuracy, we modified the RAND/UCLA algorithm to exclude nongeriatric fall injuries (eg, fall from scaffolding) and specific injury diagnoses without e-codes that we have found in other research to be inaccurately correlated with fall injury reporting.

These limitations notwithstanding, our findings may be of interest to hospitals and, more broadly, policy makers aiming to address the public health challenge of falls. Our findings suggest a motivation for hospitals to also assist with posthospital transitional safety efforts, directing at-risk patients to the appropriate postdischarge care settings, to build on policy makers’ previous efforts recognizing and addressing the burden and costs of falls.^7,8,9^ Transitional fall prevention efforts may prove economically efficient as hospitals aim to identify additional ways to avoid readmissions in the context of plateauing national readmission rates.^24,40^ The economic case may be strengthened because cost estimates for preventing a fall (approximately $300 per individual) are similar to those of widely used readmission prevention programs ($100-$200 per individual).^41,42^ The cost-effectiveness of several existing fall prevention interventions has been previously established.^43,44,45,46,47^ Targeting of patients at risk for falls, which is already routinely done in inpatient settings,^48^ can constrain spending; conversely, it remains difficult to identify patients at greatest risk for a readmission.^49^

A transitional fall-prevention model should build on the principles of existing care transition interventions. This endeavor must leverage both hospital-side and posthospital efforts. First, mobility should be encouraged and measured during the hospital stay. Second, patient and family guidance for managing fall risk and physical activity after discharge should be part of the discharge process. Third, safe mobility and multifactorial (eg, medication adjustment, physical therapy referrals for home safety checks and strength and balance training) fall prevention interventions should be instituted following discharge.^50,51^ Patients are likely to benefit from improved confidence in their ability to manage fall risks through such support.^22,27,28,52^

## Conclusions

In all, these findings suggest previously unexplored and potentially cost-saving avenues for hospitals and patients to benefit from improved inpatient and transitional fall prevention practices. Further work in this area could examine the costs along with outcomes of care, by postacute discharge disposition, of high-risk patients who experience a fall-related readmission. These could include how and whether cognitive impairment and index FRIs are jointly associated with FRI readmissions and spending. Variation in outcomes and spending for complex patients may suggest that higher quality of postacute management of functional and cognitive impairments in nursing facilities vs home care comes at great expense.^35^ Therefore, attention to improving instrumental support for patients transitioning from hospital to home may be of heightened priority. It will also be important to explore the potential for negative spillovers of Medicare policies for fall prevention. To the extent that hospital efforts prevent “never events” during the index admission by encouraging risk-averse and sedentary behavior of hospitalized patients, the postdischarge recovery may be slower, engendering unnecessary risk. Adequately addressing heightened postdischarge fall among functionally and cognitively impaired older adults will likely require increased attention to the risks and benefits of enhanced activity and rehabilitation strategies within and across care settings.

## Notes

Supplement. **eTable 1.** Exclusion Criteria for Eligible Index Cohort, Overall and by Clinical Condition Cohort **eTable 2.** Leading Clinical Classification Software (CCS) Categories for Index Fallers and Non-Fallers, 2013-2014 **eTable 3.** Clinical Characteristics of Older (≥65) Medicare Beneficiaries Overall and by Fall Injury Status for U.S. Hospital Discharges, 2013-2014 **eTable 4.** Unplanned 30-Day All-Cause Readmission Prevalence Among Older (≥65) Medicare Beneficiaries by Acute Geriatric Cohort, Across Five Clinical Index Discharge Cohorts, Overall and by Fall Injury Status, 2013-2014 **eTable 5.** Percentage of 30-Day Unplanned All-Cause Readmissions of Older (≥65) Medicare Beneficiaries Overall and Among FRI and Cognitive Impairment Index Cohorts, by Index Clinical Condition, 2013-2014
