# Supplementary material for: Posthospital Fall Injuries and 30-Day Readmissions in Adults 65 Years and Older
Source: JAMA Netw Open. 2019 May 24;2(5):e194276. doi: 10.1001/jamanetworkopen.2019.4276 (PMC6632136; doi:10.1001/jamanetworkopen.2019.4276)
Supplement: Supplement. — eTable 1. Exclusion Criteria for Eligible Index Cohort, Overall and by Clinical Condition Cohort eTable 2. Leading Clinical Classification Software (CCS) Categories for Index Fallers and Non-Fallers, 2013-2014 eTable 3. Clinical Characteristics of Older (≥65) Medicare Beneficiaries Overall and by Fall Injury Status for U.S. Hospital Discharges, 2013-2014 eTable 4. Unplanned 30-Day All-Cause Readmission Prevalence Among Older (≥65) Medicare Beneficiaries by Acute Geriatric Cohort, Across Five Clinical Index Discharge Cohorts, Overall and by Fall Injury Status, 2013-2014 eTable 5. Percentage of 30-Day Unplanned All-Cause Readmissions of Older (≥65) Medicare Beneficiaries Overall and Among FRI and Cognitive Impairment Index Cohorts, by Index Clinical Condition, 2013-2014 [file jamanetwopen-2-e194276-s001.pdf]

Supplementary Online Content

Hoffman GJ, Liu H, Alexander NB, Tinetti M, Braun TM, Min LC. Posthospital fall injuries and 30-day readmissions in adults 65 years and older. *JAMA Netw Open*. 2019;2(5):e194276. doi:10.1001/jamanetworkopen.2019.4276

eTable 1. Exclusion Criteria for Eligible Index Cohort, Overall and by Clinical Condition Cohort

eTable 2. Leading Clinical Classification Software (CCS) Categories for Index Fallers and Non-Fallers, 2013-2014

eTable 3. Clinical Characteristics of Older (≥65) Medicare Beneficiaries Overall and by Fall Injury Status for U.S. Hospital Discharges, 2013-2014

eTable 4. Unplanned 30-Day All-Cause Readmission Prevalence Among Older (≥65) Medicare Beneficiaries by Acute Geriatric Cohort, Aross Five Clinical Index Discharge Cohorts, Overall and by Fall Injury Status, 2013-2014

eTable 5. Percentage of 30-Day Unplanned All-Cause Readmissions of Older (≥65) Medicare Beneficiaries Overall and Among FRI and Cognitive Impairment Index Cohorts, by Index Clinical Condition, 2013-2014

This supplementary material has been provided by the authors to give readers additional information about their work.

eTable 1. Exclusion Criteria for Eligible Index Cohort, Overall and by Clinical Condition Cohort

		n		Percent removed (%)
1. Medicare population ≥65 years		10,111,157		
2. Exclude admissions with in-hospital death		387,949		3.8
3. Exclude admissions transferred to another acute care facility		183,576		1.8
4. Exclude admissions with less than 30 days follow-up from discharge date		821,527		8.1
5. Exclude admissions discharged against medical advice		45,159		0.5
6. Exclude admissions for primary psychiatric diagnoses		79,862		0.8
7. Exclude admissions for rehabilitation		22,053		0.2
8. Exclude admissions of cancer		187,508		1.9
**Final Cohort**		**8,382,074**		**17.1**
		n		Percent total (%)
Identify admissions for **Medicine** (Cohort 1)		3,561,772		42.5
Identify admissions for a major **Surgery** (Cohort 2)		2,070,888		24.7
Identify admissions for **Cardiorespiratory** (Cohort 3)		1,344,038		16.0
Identify admissions for **Cardiovascular** (Cohort 4)		887,879		10.6
Identify admissions for **Neurology** (Cohort 5)		517,497		6.2

eTable 2. Leading Clinical Classification Software (CCS) Categories for Index Fallers and Non-Fallers, 2013-2014

Top 10 CCS in Medicine[a]				
**Fallers**			**Non-Fallers**	
N = 306,318			N = 3,255,454	
CCS	Category Name		CCS	Category Name
231	Other fractures		2	Septicemia (except in labor)
2	Septicemia (except in labor)		159	Urinary tract infections
159	Urinary tract infections		157	Acute and unspecified renal failure
245	Syncope		153	Gastrointestinal hemorrhage
229	Fracture of upper limb		55	Fluid and electrolyte disorders
157	Acute and unspecified renal failure		197	Skin and subcutaneous tissue infections
239	Superficial injury; contusion		237	Complication of device; implant or graft
197	Skin and subcutaneous tissue infections		145	Intestinal obstruction without hernia
230	Fracture of lower limb		146	Diverticulosis and diverticulitis
226	Fracture of neck of femur (hip)		238	Complications of surgical procedures or medical care
**Top 10 CCS in Cardiorespiratory[a]**				
**Fallers**			**Non-Fallers**	
N = 35,298			N = 1,308,740	
CCS	Category Name		CCS	Category Name
122	Pneumonia (except that caused by tuberculosis or sexually transmitted disease)		108	Congestive heart failure; nonhypertensive
108	Congestive heart failure; nonhypertensive		122	Pneumonia (except that caused by tuberculosis or sexually transmitted disease)
127	Chronic obstructive pulmonary disease and bronchiectasis		127	Chronic obstructive pulmonary disease and bronchiectasis
131	Respiratory failure; insufficiency; arrest (adult)		131	Respiratory failure; insufficiency; arrest (adult)
103	Pulmonary heart disease		103	Pulmonary heart disease

128		Asthma		128		Asthma
125		Acute bronchitis		125		Acute bronchitis
				56		Cystic fibrosis
**Top 10 CCS in Cardiovascular**[a]						
**Fallers**				**Non-Fallers**		
N = 31,656				N = 856,223		
CCS		Category Name		CCS		Category Name
106		Cardiac dysrhythmias				106 Cardiac dysrhythmias
117		Other circulatory disease				100 Acute myocardial infarction
100		Acute myocardial infarction				101 Coronary atherosclerosis and other heart disease
105		Conduction disorders				102 Nonspecific chest pain
102		Nonspecific chest pain				117 Other circulatory disease
101		Coronary atherosclerosis and other heart disease				114 Peripheral and visceral atherosclerosis
114		Peripheral and visceral atherosclerosis				105 Conduction disorders
96		Heart valve disorders				97 Peri-; endo-; and myocarditis; cardiomyopathy (except that caused by tuberculosis or sexually transmitted disease)
97		Peri-; endo-; and myocarditis; cardiomyopathy (except that caused by tuberculosis or sexually transmitted disease)				96 Heart valve disorders
107		Cardiac arrest and ventricular fibrillation				115 Aortic; peripheral; and visceral artery aneurysms
**Top 10 CCS in Neurology**[a]						
**Fallers**				**Non-Fallers**		
N = 75,917				N = 441,580		
CCS		Category Name		CCS		Category Name
233		Intracranial injury				109 Acute cerebrovascular disease
109		Acute cerebrovascular disease				112 Transient cerebral ischemia

95		Other nervous system disorders		95		Other nervous system disorders
83		Epilepsy; convulsions		83		Epilepsy; convulsions
112		Transient cerebral ischemia		81		Other hereditary and degenerative nervous system conditions
81		Other hereditary and degenerative nervous system conditions		113		Late effects of cerebrovascular disease
227		Spinal cord injury		110		Occlusion or stenosis of precerebral arteries
79		Parkinson`s disease		85		Coma; stupor; and brain damage
113		Late effects of cerebrovascular disease		111		Other and ill-defined cerebrovascular disease
85		Coma; stupor; and brain damage		79		Parkinson`s disease
**Top 10 CCS in Surgical**[b]						
**Fallers**				**Non-Fallers**		
N = 297,208				N = 1,773,680		
CCS		Category Name		CCS		Category Name
146		Treatment; fracture or dislocation of hip and femur		152		Arthroplasty knee
222		Blood transfusion		222		Blood transfusion
153		Hip replacement; total and partial		153		Hip replacement; total and partial
147		Treatment; fracture or dislocation of lower extremity (other than hip or femur)		231		Other therapeutic procedures
148		Other fracture and dislocation procedure		54		Other vascular catheterization; not heart
161		Other OR therapeutic procedures on bone		61		Other OR procedures on vessels other than head and neck
145		Treatment; fracture or dislocation of radius and ulna		3		Laminectomy; excision intervertebral disc

eTable 3. Clinical Characteristics of Older (≥65) Medicare Beneficiaries Overall and by Fall Injury Status for U.S. Hospital Discharges, 2013-2014

	Overall		Fallers		Non-Fallers		*p-val* *
	n = 8,382,074		*n* = 746,397		*n* = 7,635,677		
Cohort (%)							
Medicine (*n*= 3,561,772)	42.5		41.0		42.6		<.001
Cardiorespiratory (*n*= 1,344,038)	16.0		4.7		17.1		
Cardiovascular (*n*= 887,879)	10.6		4.2		11.2		
Neurology (*n*= 517,497)	6.2		10.2		5.8		
Surgical (*n*= 2,070,888)	24.7		39.8		23.2		

* Compares fallers and non-fallers. Chi-square test is used to compare proportions.

Note: Falls were assessed at the index discharge.

eTable 4. Unplanned 30-Day All-Cause Readmission Prevalence Among Older (≥65) Medicare Beneficiaries by Acute Geriatric Cohort, Across Five Clinical Index Discharge Cohorts, Overall and by Fall Injury Status, 2013-2014

	Overall	Index FRI Acute Geriatric Cohort			Index Cognitive Impairment Acute Geriatric Cohort		
		Yes	No		Yes	No	
	n = 8,382,074	*n* = 746,397	*n* = 7,635,677	*p-val*	*n* = 1,367,759	*n*=7,014,315	*p-val*
By Cohort (%)							
Medicine (*n*= 3,561,772)	16.0	13.4	16.3	<.001	16.1	16.0	0.10
Cardiorespiratory (*n*= 1,344,038)	18.5	19.0	18.5	0.019	18.1	18.6	<.001
Cardiovascular (*n*= 887,879)	12.7	13.8	12.7	<.001	15.5	12.3	<.001
Neurology (*n*= 517,497)	12.3	13.4	12.1	<.001	13.4	12.0	<.001
Surgical (*n*= 2,070,888)	10.2	11.4	9.9	<.001	15.2	9.7	<.001

eTable 5. Percentage of 30-Day Unplanned All-Cause Readmissions of Older (≥65) Medicare Beneficiaries Overall and among FRI and Cognitive Impairment Index Cohorts, by Index Clinical Condition, 2013-2014

	**Medicine** % (Rank)	**Surgery** % (Rank)	**Cardio-respiratory** % (Rank)	**Cardio-vascular** % (Rank)	**Neurology** % (Rank)
Overall [a]	5.5 (**3rd**) N = 31,161 (570,530)	4.2 (**4th**) N = 8,806 (210,146)	4.4 (**7th**) N = 10,957 (248,966)	4.4 (**4th**) N = 4,984 (112,685)	7.9 (**3rd**) N = 5,046 (63,635)
FRI Index Cohort [b]	12.1 (**1st**) N = 4,962 (41,111)	7.2 (**2nd**) N = 2,429 (33,913)	8.6 (**3rd**) N = 576 (6,708)	9.6 (**2nd**) N = 417 (4,368)	15.0 (**1st**) N = 1,531 (10,201)
Cognitive Impairment Cohort [c]	7.2 (**2nd**) N = 9,049 (126,510)	6.3 (**2nd**) N = 1,583 (25,189)	5.8 (**6th**) N = 1,987 (34,217)	6.7 (**4th**) N = 1,029 (15,421)	9.5 (**2nd**) N = 1,614 (17,014)

[a] *n*=8,382,074; [b] *n*=746,397; [c] *n*=1,367,759
